# The Levels of Tumor Necrosis Factor-Alpha and Interleukin-6 in Patients with Isolated Coronary Artery Ectasia

**DOI:** 10.1155/2009/106145

**Published:** 2009-06-17

**Authors:** Mustafa Aydin, Ishak Ozel Tekin, Sait Mesut Dogan, Nesligul Yildirim, Mehmet Arasli, Muhammet Rasit Sayin, Ziyaettin Aktop

**Affiliations:** ^1^Department of Cardiology, School of Medicine, Zonguldak Karaelmas University, Kozlu, 67600 Zonguldak, Turkey; ^2^Department of Immunology, Zonguldak Karaelams Univerisity, Kozlu, 67600 Zonguldak, Turkey

## Abstract

*Background/Aim*. Coronary artery ectasia (CAE) is considered as a variant of atherosclerosis. Tumor necrosis factor-alpha (TNF-*α*) and interleukin-6 (IL-6) are among the sensitive markers of systemic inflammation. The aim of this study was to evaluate the plasma levels of the cytokines; TNF-*α* and IL-6 in CAE patients. *Methods*. Plasma concentrations of TNF-*α* and IL-6 were measured in 36 patients with CAE (28 males, mean age: 58.2 ± 12 years), and results were compared with age and sex-matched controls (n = 32) without coronary artery ectasia. TNF-*α* and IL-6 concentrations in blood were assesed by enzyme-linked immunosorbent assay (ELISA). *Results*. Baseline characteristics of the two groups were similar. TNF-*α* and IL-6 levels were significantly higher in CAE group than controls (15.6 ± 11.2 pg/mL versus 7.8 ± 3.7 pg/mL, *P* < .001, and 17.2 ± 12.6 versus 7.6 ± 2.1 *P* < .0001, resp.). *Conclusion*. CAE patients showed increases in TNF-*α* and IL-6 levels compared to the controls. This study provides evidence for alterations in the proinflamatory cytokines which suggest the involvement of the immune system in the pathophysiology of CAE. Further placebo-controlled studies are needed to evaluate the clinical significance of this increase in TNF-*α* and IL-6 levels.

## 1. Introduction

Coronary artery ectasia (CAE) has been defined as localized or diffuse dilation of the coronary arteries exceeding the 1.5 fold of normal adjacent segment in coronary angiography [1, 2]. CAE is a rare finding among coronary artery anomalies and considered to be of either congenital or acquired origin [2, 3]. Prevalence of CAE appears to rise in recent years [4]. It is estimated that 50% of CAE is related to atherosclerosis, whereas 20%–30% of cases may be due to congenital anomalies [3, 5]. 

Atherosclerosis is increasingly thought to be a chronic inflammatory disease [5–7]. Although it has been suggested that ectasia is commonly a variant of atherosclerosis, it is not known clearly why some patients with obstructive coronary artery disease (CAD) develop CAE, whereas others do not [1–3]. Tumor necrosis factor-alpha (TNF-*α*) and interleukin-6 (IL-6) are significantly associated with coronary atherosclerosis [8, 9]. Although the increase in the plasma levels of TNF-*α* and IL-6 in obstructive CAD is well known, limited data are available in CAE [10]. 

In this study, we aimed to evaluate the plasma levels of the cytokines; TNF-*α* and IL-6 in CAE patients.

## 2. Method

### 2.1. Patients

The study was designed as a case-control prospective study. The study population was selected from a series of 1820 consecutive patients who underwent coronary angiography in our hospital between January 2008 and September 2008 due to the presence of chest pain or positive or equivocal results of noninvasive screening tests for myocardial ischemia. Out of the 1820 patients, 36 consecutive patients who had angiographically normal coronary arteries with CAE (group I, 28 male, mean age: 58.2 ± 12 years) and accepted to participate to our study after giving informed consent were identified and compared with age, and sex-matched 32 consecutive participants with angiographically show normal coronary arteries without CAE (group II, 25 male, mean age: 57.2 ± 10 years). No significant difference was present between the two groups regarding the use of acetylsalicylic acid, beta blockers, nitrates, calcium-channel blockers, and statins.

Written informed consent was obtained prior to enrollment from all of the participants. The study protocol was approved by the institutional ethics committee, and the study was conducted in accordance with the Decleration of Helsinki and Good Clinical practice (GCP)/International Conference on Harmonization (ICH) guidelines. 

Subjects were excluded if they had evidence of traumatic injury, acute coronary syndrome history, obstructive CAD, left ventricular dysfunction, left ventricular hypertrophy, cardiomyopathies, congenital heart disease, valvular heart disease, any abnormality in thyroid function test, arrhythmias, clinically unstable medical illness such as hepatic or renal impairment; a history of seizure, head trauma, or stroke, active infection, allergy, active inflammation, collagen vascular disease, cancer, and any other primary disease interfering with immune functions.

### 2.2. Coronary Angiograms and Immunoassay

In all patients, coronary angiography was performed using the Philips Angioscop X-ray (Integris BH 5000, Philips Medical Systems, Best, The Netherlands) using standard Judkins technique. Two experienced cardiologists, unaware of the patients clinical characteristics and biochemical results, reviewed all of the angiographic images. We used iohexol (Omnipaque, Nycomed Ireland, Cork, Ireland) as contrast agent during coronary angiography in the study patients.

A coronary diameter index was defined for each segment as the coronary diameter divided by the body surface area (BSA). A coronary segment with a diameter index ≥1.5 fold of the control group was defined as ectatic. When there was no identifiable adjacent normal segment, the mean diameter of the corresponding coronary segment in the control group served as normal values. 

Blood samples of all individuals were taken 24 hours after coronary angiography from an antecubital vein. Blood samples were centrifuged immediately at 250 g for 10 minutes and stored at +4°C until assay. Triglyceride, total cholesterol, low-density lipoprotein (LDL) cholesterol, and high-density lipoprotein cholesterol concentrations were measured by automated chemistry analyzer (Aeroset, Abbott, Ill, USA) by using commercially available kits. 

Ten mL of heparinized venous blood were collected with the plastic tubes from subjects at 08.00 a.m. Blood samples were santrifuged at 3000 rpm (rotor diameter: 16 cm) and preserved at −80°C. Analyses were performed by the immunologists, who were blinded to the condition of the samples. IL-6, TNF-*α* enzyme-linked immunosorbent assay (ELISA) kits were purchased by Biosource International Inc. (Camarillo, Calif, USA) and used according to the recommendations of the manufacturer. The minimum detectable doses of TNF-*α* and IL-6 are 1.1 pg/mL, 2.2 pg/mL, respectively. There was no cross-reactivity with other cytokines. All samples were assayed in duplicate. 

### 2.3. Statistical Analysis

Statistical analysis was performed by using SPSS for windows 11.0 (Chicago, Ill, USA). Continuous variables were expressed as mean ± SD, and categorical variables were expressed as percentage. Comparison of categorical and continuous variables between groups was performed by using *x* ^2^ test and Student's *t*-test or Mann-Whitney U-test. The correlation between TNF-*α* and IL-6 levels and the number of ectasic vessels was assessed by the Pearson correlation, or the Spearman tests were appropriate. A *P*-value of <.05 was considered statistically significant. Shapiro Wilks test was used for the assessment of the distribution of continuous variables. All tests performed were two tailed. 

## 3. Results

Reproducibility was estimated by analyzing all recordings on 2 separate occasions (intraobserver variability); to assess interobserver variability, investigators were blinded. Intraobserver and interobserver coefficients of variation averaged 5%.

Baseline demographic, clinical, and angiographic characteristics of the two groups were similar (Table 1). TNF-*α* and IL-6 levels were significantly higher in CAE group than controls (15.6 ± 11.2 pg/mL versus 7.8 ± 3.7 pg/mL, *P* < .001, and 17.2 ± 12.6 versus 7.6 ± 2.1 *P* < .0001, resp.) (Table 2). Besides no correlation was found between the TNF-*α*, IL-6 levels and the number of ectatic vessels (*r* = 0.012, and *r* = 0.138, resp., *P* > .05).

## 4. Discussion

The main finding of the present study was that patients with isolated CAE had significantly higher TNF-alfa and IL-6 levels compared to control subjects with angiographically normal coronary arteries.

CAE is a well-recognized angiographic finding of abnormal coronary dilatation, and detected in 0.3%–5.3% of consecutive angiographic studies [11]. The gold standard for diagnosis of this type of ectasia is coronary angiography, which provides information about the possible size, sample, location, and number of ectasias. In fact, CAE is traditionally considered as a variant of coronary atherosclerosis. Clinically, it predisposes to adverse coronary events like vasospasm, thrombosis, and dissection [1]. In some studies, coronary aneurysms have also been strongly associated with an increased risk myocardial infarction [12]. Angina pectoris is a frequent complaint of patients with isolated CAE. Although many etiologies of CAE have been reported, the common coexistence with CAD has raised the idea that CAE may be a variant of CAD. Individual case reports have shown that isolated CAE may be a cause of silent myocardial ischemia [13].

In this study, we found significantly higher levels of IL-6 in CAE patients when compared to control subjects with normal coronary arteries. Tokgozoglu et al. found similar results with our findings [10]. We suggest that this inflammatory state in the CAE patiens is responsible for higher TNF-*α* and IL-6 levels in CAE. 

A recently published investigation reported that coronary flow reserve is significantly reduced in patients with CAE and put forward that microvascular dysfunction may be the underlying cause of exercise-induced myocardial ischemia [14].

Inflammation is a protect mechanism of the body against infectious agents and injury. There is now a well-known common aggreement that atherosclerosis is an inflammatory disorder of the vessels. Although cause and pathways of atherosclerotic process are probably multiple and different in various clinical settings, the data showed that an inflammatory process was involved in all stages of atherosclerosis [15] including coronary spasm, delayed coronary flow, coronary microvessel dysfunction, silent myocardial ischemia, and restenotic process [16, 17]. As a results of these data, the idea of chronic inflammation as one of the key factors involved in atherosclerosis has come out and broaden into a new area of atherosclerotic diseases.

A few recent data have also emphasized that CAE is associated with systemic inflammatory response exposed by inreased inflammatory cytokines and C-reactive protein (CRP). Recently, a study indicated a cytokine-induced tissue inflammation in the pathogenesis of abdominal aortic aneurysms, and it has been documented that circulating IL-6 levels increase in these patients [18]. Tokgozoglu et al. [10] studied with CAE, and found that serum IL-6 levels were significantly higher in patients with CAE compared to controls. Our results confirmed these results. We found higher levels of IL-6 in CAE patients. 

Yildirim et al. [19] studied the CAE and found that mean fluorescence intensity of CD45 and CD11b on the monocyte surface of patients with CAE was significantly higher when compared to controls. They speculated that increased levels of cellular adhesion molecules in patients with CAE may be an indicator of endothelial activation and inflammation and are likely to be in the causal pathway leading to coronary artery ectasia. Turhan et al. [20] demonstrated that the levels of CRP, a specific marker of chronic inflammation, were significantly higher in patients with isolated CAE. A recently published study showed that patients with isolated CAE have raised levels of plasma soluble intercellular adhesion molecule-1 (ICAM-1), vascular cell adhesion molecule-1 (VCAM-1), and E-selectin in comparison with patients with obstructive CAD without CAE and subjects with normal coronary arteries [21]. In addition, Turhan et al. evaluated CRP levels as a specific marker of inflammation in patients with CAE. They showed that increased levels of CRP were detected in patients with isolated CAE [22]. All these data indicate the presence of a chronic inflammation in the coronary circulation of the patients with CAE. Accordingly, CAE has been suggested to be a destructive inflammatory lesion of the vascular wall. 

Briefly, the approving of the importance of inflammation in atherosclerosis has both theoretical and practical clinical implications. The data have demonstrated that CAE is associated with inflammatory response exposed by increased inflammatory cytokines. Such studies may clarify the pathogenesis of CAE and direct towards new therapeutic applications. 

## 5. Conclusion

In conclusion, we found that TNF-*α* and IL-6 levels were significantly higher in CAE patients than subjects with normal coronary arteries. We think that TNF-*α* and IL-6 measurements may be a good prognostic value in CAE patients as in stenotic ones, suggesting that vascular wall inflammation may play a role in the pathogenesis of CAE. Further placebo-controlled studies are needed to evaluate the clinical significance of increases in these markers.

## Figures and Tables

**Table 1:** Clinical and coronary angiographic characteristics of patients with isolated coronary artery ectasia and controls.

	CAE (*n* = 36)	Controls (*n* = 32)	*P*
Age (mean ± SD)(year)^(a)^	58.2 ± 12	57.2 ± 10	>.05
Sex (male/female)^(a)^	28/8	25/7	>.05
Systolic blood pressure (mm Hg)	122 ± 18	118 ± 20	>.05
Diastolic blood pressure (mm Hg)	74 ± 8	75 ± 9	>.05
Diabetes mellitus^(a)^	21	26	>.05
Hypertension^(a)^	18	13	>.05
Total cholesterol (mg/dL)	182 ± 30	177 ± 33	>.05
HDL-cholesterol (mg/dL	40 ± 9	41 ± 11	>.05
LDL-cholesterol (mg/dL)	106 ± 8	105 ± 7	>.05
Triglycerides (mg/dL)	125 ± 35	119 ± 37	>.05
Smoking^( a)^	17	12	>.05
Family history^(a)^	25	24	>.05
Aspirin^(a)^	33 (92%)	28 (87%)	>.05
Nitrate^(a)^	16 (44%)	12 (38%)	>.05
Beta blockers^(a)^	28 (77%)	24 (75%)	>.05
Angiographic findings of ectasias			
Distribution of ectasia			
LAD	20 (56%)	—	—
LCx	24 (66%)	—	—
RCA	15 (42%)	—	—
Number of involved vessels		—	—
One-vessel	14 (38%)	—	—
Two-vessel	17 (47%)	—	—
Three-vessel	4 (11%)	—	—

Number of ectasic segments (mean ± SD)	(3.4 ± 1.2)	—	—

CAE: coronary artery ectasia; LAD: left anterior descending artery; LCx: left circumflex artery; RCA: right coronary artery; ^(a)^Chi square.

**Table 2:** TNF-*α* and IL-6 levels in the patients with CAE and control group.

	CAE (*n* = 36)	Controls (*n* = 32)	*P*
TNF-*α* (pg/mL)	15.6 ± 11.2	7.8 ± 3.7	<.001
IL-6 (pg/mL)	17.2 ± 12.6	7.6 ± 2.1	<.001

CAE: coronary artery ectasia, TNF-*α* tumor necrosis factor-alpha, IL-6; and interleukin-6.
